# Inhibition of iNKT Cells by the HLA-G-ILT2 Checkpoint and Poor Stimulation by HLA-G-Expressing Tolerogenic DC

**DOI:** 10.3389/fimmu.2020.608614

**Published:** 2021-01-11

**Authors:** Ching-Lien Wu, Julien Caumartin, Giada Amodio, François Anna, Maria Loustau, Silvia Gregori, Pierre Langlade-Demoyen, Joel LeMaoult

**Affiliations:** ^1^ CEA, DRF-Francois Jacob Institute, Research Division in Hematology and Immunology (SRHI), Saint-Louis Hospital, Paris, France; ^2^ Université de Paris, IRSL, UMRS 976, Paris, France; ^3^ Invectys, Paris, France; ^4^ San Raffaele Telethon Institute for Gene Therapy (SR-TIGET), IRCCS San Raffaele Scientific Institute, Milan, Italy

**Keywords:** Human Leucocyte Antigen G, Natural Killer T cells, immune regulation, tolerogenic dendritic cells, ILT2/CD85j/LILRB1

## Abstract

Invariant Natural Killer T (iNKT) cells are a small and distinct population of T cells crucial in immunomodulation. After activation by alpha-GalactosylCeramide (αGC), an exogenic glycolipid antigen, iNKT cells can rapidly release cytokines to enhance specific anti-tumor activity. Several human clinical trials on iNKT cell-based anti-cancer are ongoing, however results are not as striking as in murine models. Given that iNKT-based immunotherapies are dependent mainly on antigen-presenting cells (APC), a human tolerogenic molecule with no murine homolog, such as Human Leucocyte Antigen G (HLA-G), could contribute to this discrepancy. HLA-G is a well-known immune checkpoint molecule involved in fetal-maternal tolerance and in tumor immune escape. HLA-G exerts its immunomodulatory functions through the interaction with immune inhibitory receptors such as ILT2, differentially expressed on immune cell subsets. We hypothesized that HLA-G might inhibit iNKT function directly or by inducing tolerogenic APC leading to iNKT cell anergy, which could impact the results of current clinical trials. Using an ILT2-transduced murine iNKT cell line and human iNKT cells, we demonstrate that iNKT cells are sensitive to HLA-G, which inhibits their cytokine secretion. Furthermore, human HLA-G^+^ dendritic cells, called DC-10, failed at inducing iNKT cell activation compared to their autologous HLA-G^‒^ DCs counterparts. Our data show for the first time that the HLA-G/ILT2 ICP is involved in iNKT cell function modulation.

## Introduction

Natural Killer T (NKT) cells are a subset of T cells expressing distinct αβ T cell receptor (αβTCR). Initially, co-expression of T cell and Natural Killer (NK) cell markers (CD56 or CD161) were used to identify this population, therefore named NKT cells. NKT cells are now better characterized as CD1d-dependent T cells with potent cytokine production capacity (1–3). CD1d is a MHC-class-I-like molecule that mediates the presentation of lipid or glycolipid antigens to T cells. Invariant NKT (iNKT) cells, are a small subtype of the NKT population. They recognize lipids presented by of CD1d, in particular the marine-sponge-derived alpha-galactosylceramide (αGC), and express a canonical invariant TCR α chain (Vα24Jα18 in humans and Vα14Jα18 in mice) and TCR β chains that use limited Vβ segments (Vβ11 in humans and Vβ8.2 in mice) (4).

iNKT cells play an important role in anti-tumor immunity by linking the innate and adaptive immune responses. Stimulation of iNKT cells by the CD1d-αGC complex leads to a rapid production of Th1 and Th2 cytokines (e.g. IFN-γ and IL-4) and recruitment of dendritic cells (DC), NK cells, B cells, helper T cells, and cytotoxic T cells. In mice, this capability of αGC-stimulated iNKT cells to boost cellular immune responses was strong enough to generate specific responses against tumor cells, such as the B16F10 melanoma cell line, leading to long-term tumor rejection (5–7). Besides this adjuvant effect, iNKT cells can also directly control tumor growth by cytotoxicity (8). Clinical data show that iNKT cell numbers correlate with better survival of cancer patients (9) and on the contrary, that abnormal numbers and functions of iNKT cells are associated with poor clinical outcome (10, 11). Thus, there is an increasing interest in iNKT cell-based immunotherapy strategies to treat cancer.

iNKT-based anti-tumor strategies rely, so far, on harnessing iNKT cells to optimize anti-tumor vaccination through (i) intravenous injection of αGC (12) (ii) adoptive transfer of αGC pulsed APC (13) and (iii) adoptive transfer of *ex vivo* activated iNKT cells (14, 15). Clinical trials were mainly based on infusions of either αGC-loaded APC preparations or αGC-expanded enriched iNKT, which gave promising results in mouse models (16, 17). However, unlike in murine studies, results obtained with human iNKT cells are not yet convincing (18). Given that iNKT-based immunotherapies are dependent on APC, human-specific immune checkpoint-expressing or tolerogenic APCs could dampen their activation. It was shown that intravenous injection of αGC leads to iNKT cell anergy in a PD-1/PDL-1 dependent manner. Indeed, iNKT cells functions were decreased by PD-L1/PD-L2 expressed by APCs (19).

Thus, it is possible that the striking differences observed after iNKT-based anti-tumor immunizations in mice and humans could be due to differential expression of regulatory molecules in humans and mice, including species-specific murine-only and/or human-only molecules. In this work, we investigated the possible impact of the HLA-G/Immunoglobulin-like Transcript 2 (ILT2) interaction on the function of iNKT cells.

HLA-G is a molecule involved in fetal-maternal tolerance and in tumor immune escape. This non-classical HLA class I molecule has low polymorphism, unlike classical HLA class I molecules, and presents four membrane-bound (HLA-G1 to G4) and three soluble isoforms (HLA-G5 to G7). The most common and best-characterized isoforms, HLA-G1 and HLA-G5, are non-covalently associated with β-2-microglobulin (B2M) (20, 21). HLA-G physiological expression is tissue-restricted, mainly to trophoblast, thymus, cornea, and mesenchymal stem cells in physiological conditions. However, HLA-G can be induced under pathological conditions such as viral diseases, inflammatory disorders, transplantation and cancer (22).

HLA-G immuno-modulatory functions on all immune cell subsets are exerted through specific binding to inhibitory receptors. ILT2/CD85j/LILRB1 is one of the known HLA-G receptors, which is expressed on various proportions of monocytes, DC, B, NK, and T cells (23). ILT2 has four tandem Ig-like extracellular domains and four immunoreceptor tyrosine-based inhibitory receptor motifs (ITIM) in its cytoplasmic tail. In the case of T and NK cells, HLA-G:ILT2 interaction was reported to inhibit alloproliferation (24–28), alter cytokine secretion (25, 29–32), and inhibit the antigen-specific cytolytic functions of cytotoxic T lymphocytes (CTLs) (33, 34), uterine NK cells and peripheral blood NK cells (35, 36).

HLA-G-expressing tumor cells or high levels of HLA-G in plasma have been reported in numerous types of cancers and associated with higher grade and worse prognosis (22, 37–41). Indeed, HLA-G plays the role of an immune escape mechanism through inhibition of anti-tumor effectors, alteration of cytokine expression patterns (14, 37, 38), and generation of regulatory cells (39, 40). Furthermore, tumors can induce HLA-G expression by other cells such as tolerogenic APCs (e.g. DC-10 cells), leading to T cell anergy and induction of regulatory T cells (42, 43). Interestingly, ILT2 expression has also been associated with tumor immune escape (44). Thus, HLA-G:ILT2 is a potent immune checkpoint and constitutes a potential new target in anti-tumor therapies.

iNKT cells are related to both NK and T cells since they are T cells expressing markers mostly associated with NK cells, in particular inhibitory receptors (45). Since human NK cells and classical T cells were shown to be inhibited by HLA-G through ILT2 receptor expression, we reasoned that iNKT cells could be sensitive to HLA-G that would be expressed by the tumor cells themselves or by antigen-presenting cells such as the recently discovered HLA-G-positive DC-10 tolerogenic DC subset. Our results show that this is indeed the case. As HLA-G is known to be present in the tumor microenvironment, it could inhibit iNKT cell reactivity to αGC and impair the effectiveness of the iNKT cell-based immune therapy.

## Materials and Methods

### Human PBMC Isolation

Blood in ETDA tubes or from plateletpheresis residues was collected at the French Blood Center (EFS, Saint Louis Hospital, France) from healthy donors with informed consent. Human PBMC from healthy donors were used for ILT2 analysis, and for CD14^+^ monocyte and iNKT cells isolation. PBMC were isolated by density gradient separation using either Ficoll (Sigma-Aldrich) or Leucosep™ tube (Grenier Bio-One), washed twice in 0.9% NaCl (Versylène^®^ Fresenius), and counted using trypan blue dye in KOVA counting slides (Fisher Scientific). Viability was always >90%. Processing was completed within less than 10 h for all sample specimens.

### Flow Cytometry Analysis

Surface markers and intracellular cytokines were analyzed by flow cytometry. Labeling steps were performed by using between 0.2–3×10^6^ cells per test according to the experimental requirements in either FACS tubes or 96-well U-bottom plates. Washing steps were performed with PBS followed by centrifugation at 800 g for 1 min. Surface labeling was carried out by blocking Fc receptors, either with anti-mouse CD16/32 (eBioscience) for murine cells or human immunoglobulin G (Sigma) for human samples, for 5 min at room temperature, followed by antibody incubation for 30 min at 4°C in the dark using concentrations according to the manufacturer’s instructions. Cells were fixed in 200 µl PBS containing 1% formaldehyde and acquired within 24 h after two washing steps. To detect intracellular cytokines, surface-labeled cells were fixed with 4% PFA for 10 min at 4°C, and then permeabilized for the intracellular staining with Perm/Wash solution (BD Bioscience) following manufacturer’s instruction prior to incubation with cytokine specific antibodies.

Antibodies used in flow cytometry analysis were: i) Anti-murine: CD1d-PE (clone 1B1, BD Phamingen), CD3-APC (clone REA606, Miltenyi Biotech), IL-2-FITC (clone JES6-5H4, eBioscience), and PIR-A/B-PE (clone 10-1-PIR, BD Phamingen); ii) Anti-human: CD1d-PE (clone 51.1, eBioscience), CD1d tetramer-APC (pre-loaded with and without αGC, ProImmune), CD3-eFluor450 (clone OKT3, eBioscience), CD11c-APC (clone 3.9, eBioscience), CD14-PerCP-Cy5.5 (clone 61D3, eBioscience), CD56-PE-Cy7 (clone CMSSB, eBioscience), CD86-PE-Cy7 (clone IT2.2, eBioscience), HLA-DR-FITC (clone MEM-12, Exbio), HLA-G (clone MEM-G/09, Exbio), IFNg-PerCP-Cy5.5 (clone 4S.B3, eBioscience), IL-4-APC (clone 8D4-8, eBioscience), ILT2-PE (clone HP-F1, eBioscience), CD161-APC (clone HP-3G10, eBioscience), and Va24Ja18 TCR-FITC (clone 6B11, eBioscience). The matched isotype controls were systematically used.

Acquisition was performed on either a BD FACSCanto™ II equipped with BD FACSDiva™ software (version 6.0, BD Bioscience) or a MACSQuant^®^ Analyser 10 equipped with MACSQuantify™ Analysis Software (version 2.8, Miltenyi Biotech). The PMT voltages were adjusted for each fluorescence channel using unstained cells and compensations were set using a mixture of unstained and single color stained cells with antibodies. Analyses were performed by FlowJo software (version 10, FlowJo LLC).

### ILT2 Expression on Lymphocytes

Three million freshly isolated PBMC from 14 healthy donors were used to perform ILT2 expression analysis by flow cytometry. The CD3-eFluor450, CD56-PE-Cy7, Vα24Jα18 TCR-FITC, CD1d tetramer-APC, and ILT2-PE and their matched isotype controls were used as a five-color staining. The ILT2 expression was analyzed on CD4^+^ T cells (CD3^+^CD56^‒^CD4^+^ lymphocytes), CD8^+^ T cells (CD3^+^CD56^‒^CD8^+^ lymphocytes), NK cells (CD3^‒^CD56^+^ lymphocytes), CD3^+^CD56^+^ NKT cells (CD3^+^CD56^+^ lymphocytes) and iNKT cells (CD3^+^6B11^+^ lymphocytes). Cytometry analysis was set according to the isotype controls and all results were expressed as % of ILT2^+^ among studying subsets.

### Murine iNKT and APCs

The murine NKT1.2 [Vα14i NKT cell hybridoma 1.2 in (46)], C57BL/6) and A20CD1d (CD1d-transduced A20 B cells line, ATCC, Balb/c) lines were used as iNKT and APCs, respectively. They were kindly provided by Pr. Mitchell Kronenberg (La Jolla Institute, CA, USA) and cultured in X-VIVO-10 medium (Lonza) at 37°C with 5% CO_2_.

HLA-G-expressing and ILT2–expressing stable cell lines were generated by transduction and the lentiviral particles were generated as follows: specific sequences corresponding to native ILT2 cDNA (NM_006669.6), HLA-G1 cDNA (NM_002127.5) modified K334A and K335A according to Zhao et al. (47), and human beta-2-microglobulin (hB2M) cDNA (NM_004048.2) were cloned separately into a pTrip plasmid vector by digestion/ligation after extraction by PCR with specific primers, under CMV immediate early promoter. HIV-1-derived vector particles were produced by calcium phosphate co-transfection of HEK-293T cells (ATCC) with the recombinant plasmid pTRIP, an envelope expression plasmid encoding the glycoprotein from VSV, serotype Indiana glycoprotein, and the p8.74 encapsidation plasmid. Viral stocks were titrated by real-time PCR on cell lysates from transduced HEK-293T cells and expressed as transduction unit (TU) per ml.

To generate A20CD1d‒HLA-G/hB2M and NKT1.2-ILT2 cell lines, 1×10^5^ A20CD1d or NKT1.2 cells were seeded in 12-well plate with in 500 µl of X-VIVO-10 medium and 10^6^ TU (293T) of Trip CMV-HLAG plus Trip CMV-hB2M or Trip CMV-ILT2 vectors. Cells were incubated for 1 h at 37°C and then centrifuged 1 h at 37°C 1,200 g. Afterwards, 1 ml of X-VIVO-10 medium was added and incubated at 37°C. Two weeks later, positive cells were sorted by flow cytometry using anti-HLA-G or anti-ILT2 antibodies. The expression of, HLA-G, ILT2, murine CD1d, and PIR-B were evaluated by flow cytometry before the iNKT activation assay.

### Human mDC and DC-10 Differentiation

Human CD14^+^ monocytes were isolated from fresh PBMC by positive selection using CD14 MicroBeads (Miltenyi Biotech, Germany) according to the manufacturer’s instructions. Cells were cultured in RPMI 1640 (Lonza, Italy) supplemented with 10% Fetal Calf Serum (Lonza, Italy), 2 mM L-glutamine (Lonza, Italy), and 100 U/ml penicillin/streptomycin (Lonza, Italy) at 37°C. To induce mature DC (mDC), CD14^+^ monocytes were kept in culture with 10 ng/ml rhIL-4 (R&D Systems, Minneapolis MN, USA) and 100 ng/ml rhGM-CSF (Genzyme, Seattle WA, USA) for 5 days and maturation was induced by the addition of 1 μg/ml of LPS (Sigma, CA, USA) for additional 2 days. To differentiate DC-10, CD14^+^ monocytes were kept in culture with 10 ng/ml rhIL-4, 100 ng/ml rhGM-CSF, and10 ng/ml of rhIL-10 (BD, Pharmigen, CA, USA) for 7 days. DCs were harvested and analyzed for lineage maturation makers (CD14, CD1a, CD11c, HLA-DR, and CD86), CD1d and HLA-G by flow cytometry before the iNKT activation assay.

### Human iNKT Isolation and Expansion

Human CD14^+^ monocytes and iNKT cells were isolated from fresh PBMC by positive selection using CD14 MicroBeads and anti-iNKT MicroBeads (Miltenyi Biotech, Germany), respectively, according to manufacturer’s instructions. iNKT expansion was performed as described (48) with modifications. Briefly, iNKT were co-cultured with CD14^+^ monocytes at 1:1 ratio in RPMI 1640 (Sigma) supplemented with 10% fetal bovine serum (Sigma-Aldrich), 2 mM L-glutamine (Gibco), 10 µg/ml gentamicin (Gibco), and 0.25 µg/ml fungizone (Gibco) in the presence of 20 ng/ml GM-CSF (Peprotech), and 20 ng/ml IL-4 (R&D Systems), and 100 ng/ml αGC (Cayman Chemical). Half of the medium was replaced and 20 U/ml IL-2 (Chiron, Emeryville, USA) was added every day from day 2 to day 21 in order to reach around 1×10^6^ cells. ILT2 expression was evaluated and iNKT cells were phenotypically validated by flow cytometry before the iNKT activation assay. Purity of the iNKT cell population was systematically higher than 90%. The iNKT cells used in the activation assay with DC-10 cells were isolated and maintained in culture with the presence of IL-2 and αGC till the autologous mDC and DC-10 cells were differentiated.

### iNKT Activation Assays

The APC (A20CD1d, mDC, or DC-10) and NKT cells (NKT1.2, or human iNKT cells) were co-incubated at 1:1 ratio (1×10^6^ cells/ml) in 96-well U-bottom plates for 24 and 4 h respectively for the assay of NKT1.2 cells and human iNKT cells. αGC-loaded APCs were prepared by incubating APC with 100 ng/ml of αGC in DMSO during 1 h at 37°C. Control APCs were prepared concomitantly by adding the same volume of DMSO without αGC. For blocking experiments, both APC and NKT cells were pretreated with either anti-HLA-G (functional grade, clone 87G; Exbio) or anti-ILT2 (functional grade, clone GHI/75; BioLegend) at 37°C for 1 h prior to the co-incubation. The protein transport inhibitor (eBioscience) was used at 1× to stop the cytokine release. NKT cell phenotypes and intracellular cytokines labeling was performed as described above. The expression of murine IL-2 and human IFN-γ and IL-4 were analyzed in NKT1.2 cells (CD3^+^ cells) and human iNKT cells (singlet CD3^+^6B11^+^ lymphocytes or CD3^+^CD161^+^ lymphocytes), respectively. The gates for cytokine expression were set according to the non-activated controls in each independent experiment.

### Statistics

Shapiro-Wilk normality test, one-way ANOVA, Bonferroni’s Multiple Comparison Test, and statistical plots were performed in Prism 5 software (GraphPad). P-value ≤ 0.05 was considered statistically significant.

## Results

### Human iNKT Cells Express Cell-Surface ILT2 Upon Activation

**Figure 1:**
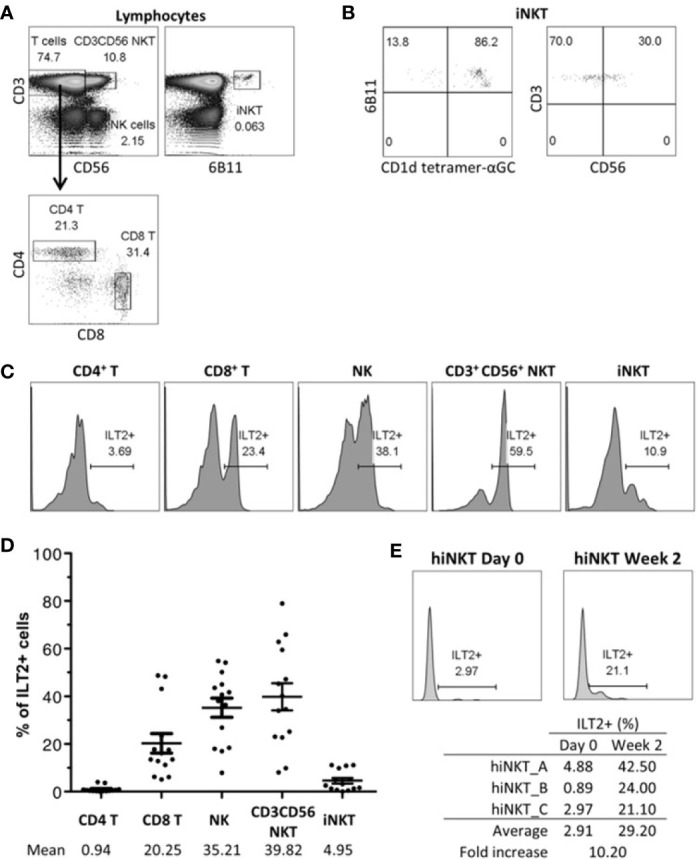
Human Invariant Natural Killer T (iNKT) cells express cell-surface Immunoglobulin-like Transcript 2 (ILT2). ILT2 expression on CD3^+^CD56^+^ NKT cells and CD3^+^6B11^+^ iNKT cells was evaluated with fresh PBMC by flow cytometry and was compared to that of CD4^+^ T, CD8^+^ T, and NK cells. **(A)** Gating strategy to identify CD4^+^ T, CD8^+^ T, NK, CD3^+^ CD56^+^ NKT, and iNKT cells from lymphocytes gated according to the FSC-SSC profile and to the CD3, CD4, CD8, CD56, and 6B11 expression. Percentage of parent population is indicated. **(B)** CD1d tetramer-αGC reactivity and CD56 expression of iNKT cells. Percentage of parent population is indicated. **(C)** ILT2 expression on different subsets of one representative blood donor. Percentage of ILT2^+^ cells is indicated. **(D)** Percentage of ILT2^+^ cells of each subpopulation from 14 healthy donors. Bars indicate mean ± SEM. **(E)** ILT2 expression on iNKT cells during expansion. iNKT cells at day 0 and 2 weeks after stimulation by CD1d-αGC and cytokines are shown. Percentage of ILT2^+^ cells is indicated.

To determine whether NKT cells can be inhibited by HLA-G, we first evaluated their ILT2 expression in comparison to autologous CD4^+^ T cells (CD3^+^CD56^‒^CD4^+^ lymphocytes), CD8^+^ T cells (CD3^+^CD56^‒^CD8^+^ lymphocytes), and NK cells (CD3^‒^CD56^+^ lymphocytes). As shown in **Figure 1A**, CD3^+^CD56^+^ NKT cells (i.e. CD3^+^CD56^+^ lymphocytes) generally represented almost 10% of peripheral lymphocytes. On the other hand, iNKT (i.e. CD3^+^6B11^+^ lymphocytes), showing double positivity for CD3 and Vα24Jα18 expression (6B11^+^) represented only 0.06% of peripheral lymphocytes. Subgating of this small subset showed them to be CD1d-αGC-reactive, but only 30% of them expressed CD56 (**Figure 1B**).

ILT2 expression by CD3^+^CD56^+^ NKT and iNKT cell subsets was then investigated in peripheral blood monocuclear cells (PBMC) of 14 healthy donors. As shown on **Figure 1C**, only a minority of iNKT expressed ILT2 at their surface. We compared ILT2 expression by CD3^+^CD56^+^ NKT and iNKT cells to those of other lymphocyte cell subsets (**Figure 1D**). Approximately 1% of CD4^+^ T cells, 20% of CD8^+^ T cells, and 35% of NK cells expressed ILT-2 receptors at their surface, which is in accordance with what has been reported (22). The CD3^+^CD56^+^ NKT cell population expressed ILT2 the most (39.82%), with values similar to those of NK cells (~35%) (**Figure 1D**). This was in sharp contrast to iNKT cells, which barely expressed ILT2 (~5%).

It is known that cell-surface ILT2 expression can be induced on T cells following activation (49). Thus, we stimulated 6B11^+^CD3^+^ iNKT cells with autologous αGC-loaded CD14^+^CD1d^+^ APCs purified from healthy donors, as previously reported (48). We showed (**Figure 1E**) that ILT2 expression is indeed induced upon iNKT cell activation, resulting in a 10-fold increase compared to unstimulated iNKT cells.

### HLA-G:ILT2 Pathway Inhibits iNKT Cell Activation in a Murine *In Vitro* Model

**Figure 2:**
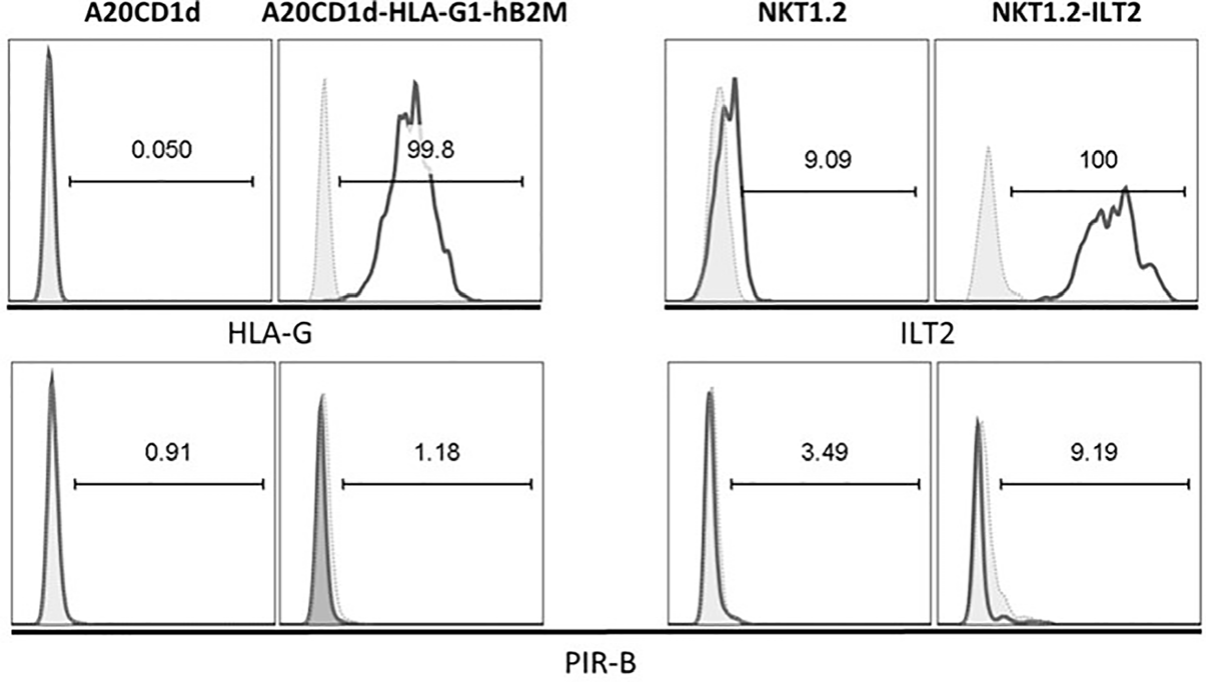
A20CD1d and NKT1.2 cells lines generated to evaluate Human Leucocyte Antigen G (HLA-G):Immunoglobulin-like Transcript 2 (ILT2) inhibitory effect. A20CD1d and NKT1.2 cells were transduced to express HLA-G and ILT2, respectively. Flow cytometry was performed to measure the expression of HLA-G, ILT2, and PIR-B. Open histograms: staining for the indicated antibodies. Shaded histograms: isotype controls. Percentage of the parent population is indicated.

Human iNKT cells represent only 0.01% to 0.1% of peripheral lymphocytes and their ILT2 expression is dependent on their activation state, reducing even further the numbers of human iNKT cells available for setting up the conditions of our functional assays. Given this and the fact that no human iNKT cell lines exist, we first developed an *in vitro* assay using murine cell lines. The murine line A20CD1d, transduced with HLA-G1 and hB2M, was used as APCs presenting αGC to the ILT2-transduced NKT1.2 effector cell line, as described in Materials and Methods. HLA-G1 expression on A20CD1d‒HLA-G1‒hB2M cells and ILT2 expression on NKT1.2-ILT2 cells are shown in **Figure 2**. Of note, no association between HLA-G1 heavy chain and murine B2M was observed, and HLA-G1 was expressed at the cell surface only in A20CD1d cells transduced with both HLA-G1 and human B2M (**Supplemental Figure 1**). Although HLA-G does not have a murine homolog, it can interact with PIR-B, the functional murine homolog of human ILT receptors (50). As can be seen in **Figure 2**, PIR-B was not expressed by either A20CD1d or NKT1.2 cell lines, ruling out any effect of HLA-G binding endogenous murine receptors, leaving transduced ILT2 as sole HLA-G receptor on NKT1.2-ILT2 cells.

To evaluate the capability of HLA-G to inhibit iNKT cells through ILT2, 24-h co-incubations between A20CD1d ± HLA-G/hB2M, and effector NKT1.2 cells ± ILT2 in the presence or absence of αGC were performed, and intracellular IL-2 expression by NKT1.2 cells was evaluated by flow cytometry (51).

**Figure 3:**
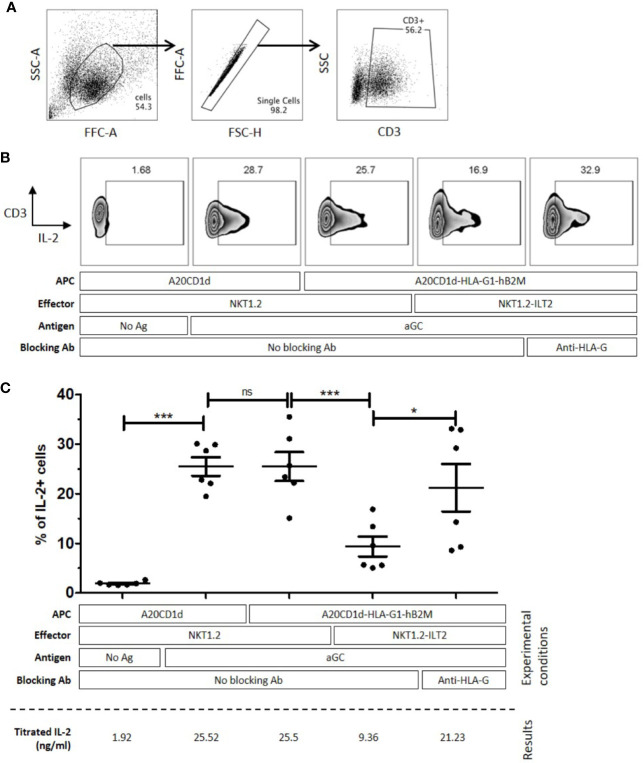
NKT1.2-ILT2 cells are inhibited through the Human Leucocyte Antigen G (HLA-G)/Immunoglobulin-like Transcript 2 (ILT2) interaction. A20CD1d or A20CD1d-HLA-G1-hB2M cells were used as Antigen Presenting Cells (APC) and NKT1.2 or NKT1.2-ILT2 cells were used as effector cells. Intracellular IL-2 expression of effector NKT cells was evaluated by flow cytometry after 24 h of co-culture with APC loaded or not with αGC as indicated. Anti-HLA-G antibody (Ab), 87G, was used to block the HLA-G/ILT2 interaction when indicated. **(A)** Gating strategy to identify NKT1.2 cells after the coculture assay. FSC-SSC profile was used to identify living cells, then the NKT1.2 cells are identified as CD3+ singlet living cells. Percentage of lymphocytes is indicated. **(B)** Intracellular IL-2 expression in NKT1.2 cells of one representative experiment. The gate of IL-2 expression is based on the no activation control in each experiment. **(C)** Six independent experiments were performed. Each bar indicates mean ± SEM. Horizontal bars indicate statistical significance (One-way ANOVA and Bonferroni *post hoc*, *p ≤ 0.05; ***p ≤ 0.001).

**Figure 3** summarizes the results obtained from six independent experiments and demonstrates that intracellular IL-2 expression was increased in NKT1.2 cells after αGC stimulation in the absence of HLA-G (p ≤ 0.001), and was significantly decreased for NKT1.2-ILT-2 cells only in the presence of HLA-G (p ≤ 0.001). Addition of a blocking anti-HLA-G antibody restored IL-2 expression upregulation (p ≤ 0.05), demonstrating that the inhibition observed was indeed due to the HLA-G:ILT2 interaction.

### HLA-G:ILT2 Pathway Inhibits Human iNKT Cell Activation

**Figure 4:**
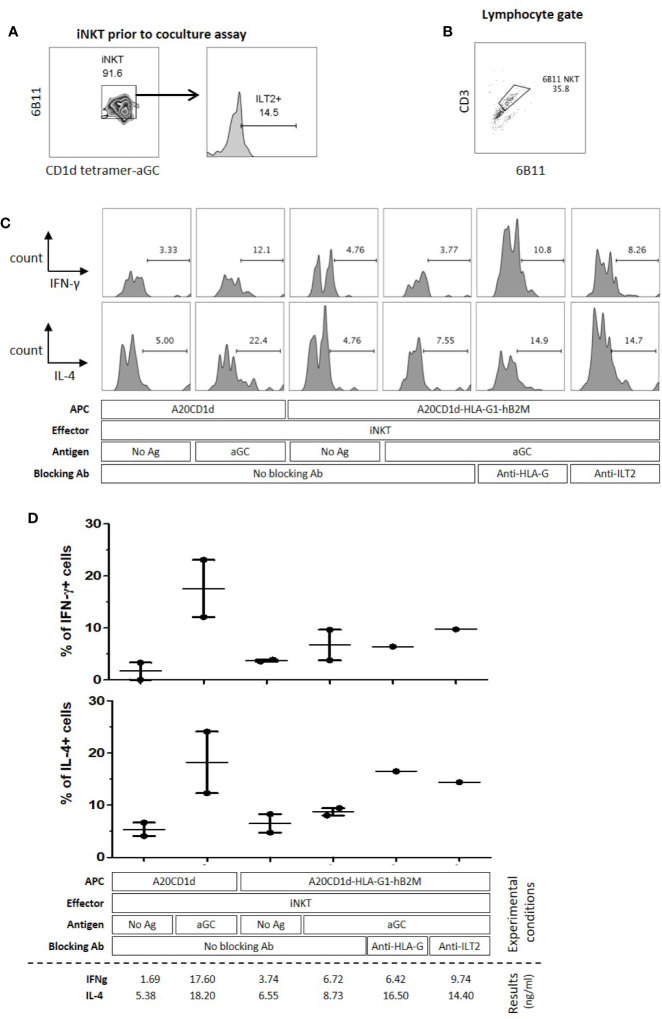
Activation of human Invariant Natural Killer T (iNKT) cells is inhibited through the Human Leucocyte Antigen G (HLA-G):Immunoglobulin-like Transcript 2 (ILT2) interaction. A20CD1d or A20CD1d-HLA-G1-B2M cells were used as Antigen Presenting Cells (APC) and human iNKT cells were used as effector cells. Intracellular IFN-γ and IL-4 expression by effector NKT cells was evaluated by flow cytometry after a 4-h co-culture with APC loaded or not with αGC as indicated. Anti-HLA-G antibody 87G, or anti-ILT2 antibody GHI/75, were used to block the HLA-G/ILT2 interaction as indicated. **(A)** Before functional assay, expanded human iNKT cells were checked using 6B11 and CD1d-tetramer-αGC. Their surface ILT2 levels were measured. **(B)** Gating strategy to identify human iNKT cells after the coculture assay with stimulator cells, gated on lymphocytes according to the FSC-SSC profile and CD3+ and 6B11+ expression. Percentage of lymphocytes is indicated. **(C)** IFN-γ (upper panel) and IL-4 (lower panel) expression levels by effector cells of one representative experiment. The gate of cytokine expression is based on the no activation control in each experiment. **(D)** Two independent experiments were performed; bar indicates mean ± SEM.

In order to evaluate whether the reactivity of human iNKT cells naturally expressing ILT2 could also be inhibited through the HLA-G:ILT2 interaction, we first stimulated human iNKT cells from healthy donors with αGC-loaded A20CD1d and A20CD1d‒HLA-G1‒hB2M cells. Indeed, it was reported that human iNKT cells can be stimulated by αGC presented in the context of murine CD1d (52). Because resting human iNKT cells barely express ILT2 as shown earlier in this work, our experiments required that ILT2 receptor expression be boosted by an *in vitro* expansion step prior to use, following published protocols (48) with modifications described in Materials and (**Figure 1E**). Two donors were used for these experiments, and results did not reach significance but only show tendency. Following amplification, 25% of iNKT cells from both donors expressed cell-surface ILT2 (data not shown). Stimulation results show that in the absence of αGC, only 1%–4% of iNKT expressed IFN-γ and 5%–7% of iNKT expressed intracellular IL-4 (**Figure 4**). Intracellular IFN-γ and IL-4 expression increased to 17.6% and 18.2% respectively when stimulated by αGC-loaded A20CD1d for 4 h, whereas intracellular IFN-γ and IL-4 expression was respectively 6.72% and 8.73% after a 4-h co-incubation with αGC-loaded A20CD1d‒HLA-G1‒hB2M cells (**Figure 4**). The presence of blocking anti-HLA-G or anti-ILT2 antibodies restored intracellular IFN-γ and IL-4 expression by iNKT cells stimulated by αGC-loaded A20CD1d‒HLA-G1‒hB2M cells, demonstrating that human iNKT inhibition was dependent on HLA-G:ILT2 interaction.

These results demonstrate that human iNKT cells are sensitive to HLA-G inhibition through ILT2 receptors engagement.

### αGC-Loaded HLA-G-Expressing Tolerogenic DC-10 Cells Do Not Activate Human iNKT Cells

**Figure 5:**
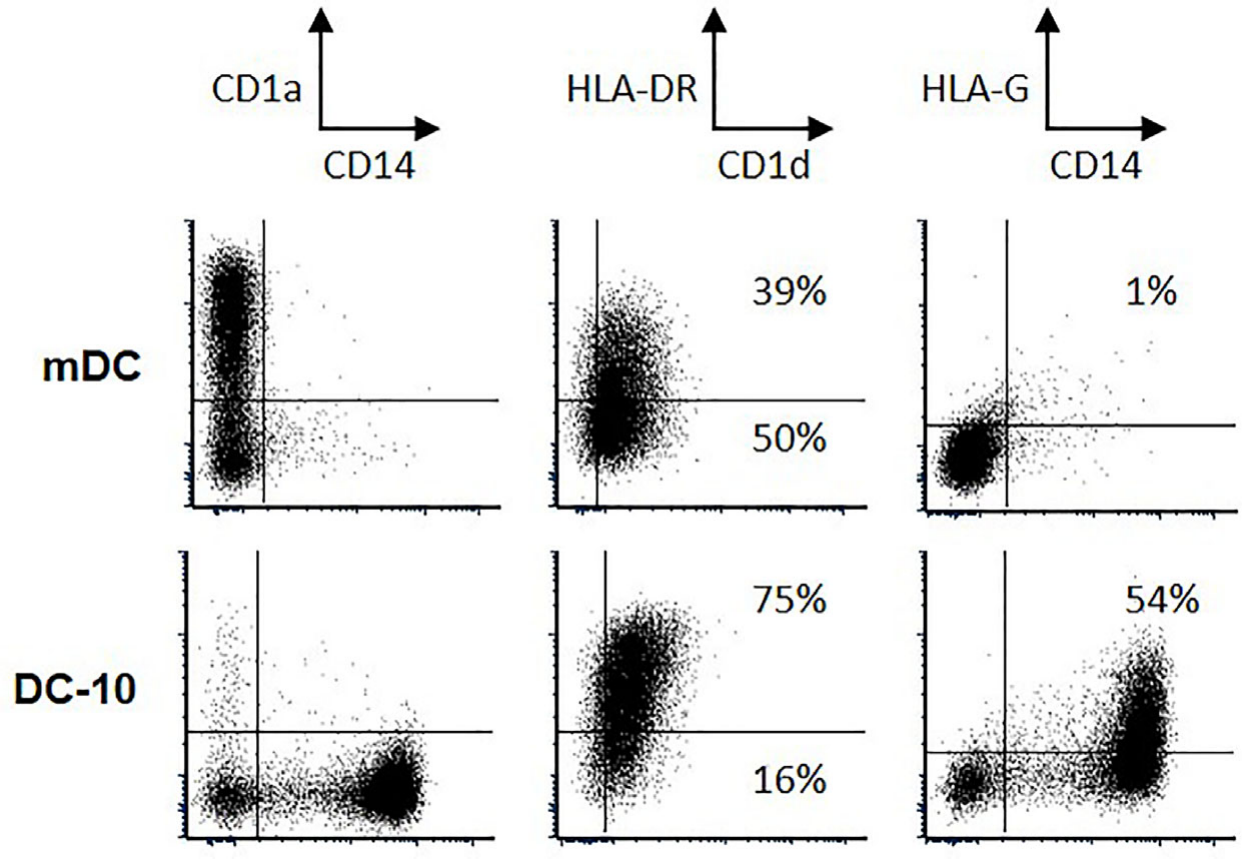
Phenotypic characteristics of mature dendritic cell (mDC) and autologous DC-10 cells. Phenotype was validated based on the expression of CD14, CD1a, CD1d, HLA-DR, and Human Leucocyte Antigen G (HLA-G); numbers indicate the percentages of positive cells for CD1d and HLA-G.

DC-10 cells are human tolerogenic DC expressing high levels of HLA-G. They are regulatory cells capable of inhibiting allogeneic responses through HLA-G and IL-10, of inducing IL-10-producing T regulatory type 1 (Tr1) cells (42, 43), and they were shown to increase in cancer patients (53, 54). Accordingly, we investigated whether tolerogenic DC-10 cells could inhibit the functions of autologous human iNKT. Human iNKT cells were stimulated with αGC-loaded autologous mDC or autologous DC-10 cells, and IFN-γ and IL-4 expression was analyzed. Freshly isolated monocytes from two healthy donors were differentiated into mDC and DC-10 cells as previously described (42), and in parallel, iNKT cells were isolated from the same donors and amplified *in vitro* in the presence of IL-2 and αGC. As expected (42, 43), DC-10 *in vitro*-differentiated from peripheral monocytes expressed cell-surface HLA-G1, while mDC barely expressed HLA-G1 (**Figure 5**). Conversely, mDC and DC-10 expressed similar cell-surface expression levels of CD1d, indicating that both cell types were capable of presenting αGC to autologous human iNKT cells.

**Figure 6:**
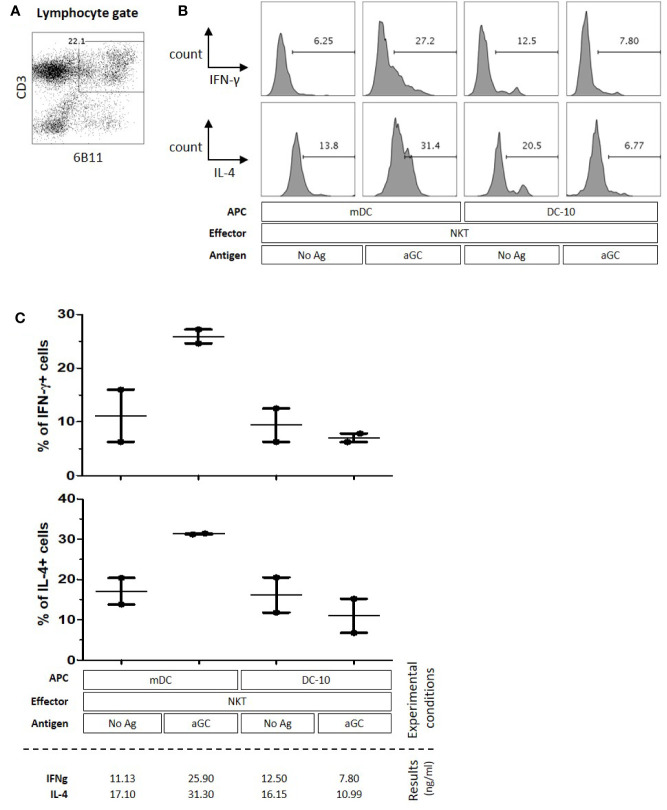
Tolerogenic DC-10 cells do not support human Invariant Natural Killer T (iNKT) cell activation. Mature Dendritic Cells (mDC) or tolerogenic DC-10 cells were used as antigen-presenting cell (APC), and autologous human iNKT cells were used as effector cells. Intracellular IFN-γ and IL-4 expression of effector cells was evaluated by flow cytometry after 72 h of co-culture with APC loaded or not with αGC antigen as indicated. **(A)** Gating strategy to identify iNKT cells after the coculture assay with stimulator cells, gated on lymphocytes according to the FSC-SSC profile and CD3^+^ and CD161^+^ expression. Percentage of lymphocytes is indicated. **(B)** IFN-γ (upper panel) and IL-4 (lower panel) expression levels by effector cells of one representative experiment. The gate of cytokine expression is based on the no activation control in each experiment. **(C)** Two independent experiments were performed; bar indicates mean ± SEM.

iNKT cells were then co-incubated for 72 h with either autologous mDC or DC-10 and their intracellular IFN-γ and IL-4 expression was evaluated by flow cytometry. It is unfortunate that we could obtained all required cell populations from two donors only, preventing the results from reaching statistical significance, although tendencies were clear: **Figure 6B** shows the results obtained for one donor, and **Figure 6C** presents data for both donors. As can be seen for both experiments combined, 11%–12% of iNKT cells expressed IFN-γ and 16%–17% expressed IL-4 when stimulated with unloaded mDC and DC-10. When iNKT cells were stimulated with αGC-loaded mDC, IFN-γ and IL-4 expression increased, to 26% and 31%, respectively (**Figure 6C**). Conversely, when iNKT cells were stimulated with αGC-loaded DC-10, no increase in IFN-γ and IL-4 expression was observed, and the proportion of iNKT cells positive for IFN-γ and IL-4 remained at or below the baseline obtained without αGC (7% and 10% respectively) (**Figure 6C**). These results indicated that HLA-G-positive αGC-loaded DC-10 cells do not seem to support human iNKT activation.

## Discussion

We sought to prove that the interaction of HLA-G with ILT2 could inhibit iNKT activation. Thus, we first evaluated the ILT2 cell-surface expression on iNKT cells. Surprisingly, although CD3^+^CD56^+^ NKT cells expressed high levels of cell-surface ILT2, the iNKT cell subset, specific for αGC presented in the context of CD1d, barely expressed ILT2 (**Figure 1D**). Indeed, ILT2 expression on resting human iNKT cells from peripheral blood was higher than that of CD4^+^ T cells but lower than that of CD8^+^ T cells or NK cells. Therefore, following these first results, it seemed unlikely that HLA-G could have any significant direct inhibitory effect on iNKT cells. However, it must be considered that even though CD4^+^ T cells express ILT2, even less so than iNKT cells, they are well known to be sensitive to HLA-G inhibition anyway (27, 28, 55). This discrepancy between weak ILT2 expression and sensitivity to HLA-G can be explained by two hypotheses: first, T cell inhibition by HLA-G may not be direct; HLA-G could act on stimulating APC or through the generation of tolerogenic APC. This mechanism is very relevant to iNKT cells since monocytes/DC that are required for αGC-induced iNKT activation constitutively express the ILT2 and ILT4 HLA-G receptors (23, 56), and are known to be inhibited by HLA-G (57–59). Second, ILT2 might not be readily present on peripheral blood resting T cells, but be upregulated upon activation. In this case, HLA-G would not act directly on resting iNKT cells, but only on already activated ones. Several studies demonstrated that ILT2 is upregulated by CD4^+^ and CD8^+^ T cells upon activation (49). In agreement with these results, our experiments showed that ILT2 was upregulated on iNKT cells in response to stimulation, and that *in vitro* expanded iNKT cells expressed cell-surface ILT2 at a level comparable to that of polyclonal NK cells (**Figure 1E**). Thus, according to our results, it seems that HLA-G direct inhibition should only impact activated iNKT cells.

To demonstrate that activated iNKT cells could be inhibited by HLA-G, we developed an *in vitro* model using a murine NKT cell line. Indeed, iNKT cells, and even more so ILT2-positive iNKT cells, represent a very small subset of peripheral lymphocytes which is difficult to isolate. Thus, to overcome this limitation, we set up HLA-G/ILT2 ICP inhibition experiments in the already described NKT1.2 vs A20CD1d model and transduced them with ILT2 and HLA-G/hB2M respectively. We showed that NKT1.2 cells rapidly expressed IL-2 in response to stimulation by αGC-loaded A20CD1d cells expressing HLA-G or not (**Figure 3**). However, IL-2 upregulation was hampered when ILT2-expressing NKT1.2-ILT2 cells were stimulated by HLA-G-expressing αGC-loaded A20CD1d-HLA-G1-hB2M cells (**Figure 3**). IL-2 expression inhibition was due to the ILT2:HLA-G1 interaction given that anti-HLA-G blocking antibodies restored IL-2 expression (**Figure 3**). We demonstrated that HLA-G could directly inhibit ILT2-expressing iNKT cells. Transduced murine cell lines may not be representative of human iNKT cells, however the human iNKT cells interaction with αGC-loaded murine CD1d could be used to study human iNKT cells inhibition by HLA-G. αGC-loaded murine A20CD1d/A20CD1d-HLA-G1-hB2M cells were used to stimulate human *in vitro*-expanded iNKT cells (**Figure 4**). In these experiments, iNKT cells had been expanded *in vitro* in order to induce ILT2 expressions sufficiently so that the ILT2^+^ population was analyzable. It is unclear whether naïve, resting iNKT could respond to HLA-G as activated iNKT did. One can hypothesize that only previously stimulated iNKT would respond to HLA-G. Those iNKT cells, recognizing specific CD1d-lipid complexes would then be akin to memory T cells and sensitive to HLA-G inhibition in recall immunization or in the context of chronic stimulation. One can also hypothesize that resting, naïve iNKT cells would be insensitive to HLA-G but only until they are fully activated and upregulate ILT2. In this case, HLA-G would not prevent the early functions of iNKT, but rather their late functions, and possibly shorten their activation.

When studying the possible effect of HLA-G:ILT2 ICP on iNKT cell activation and functions, it is necessary to consider the stimulatory cells. Indeed, in the context of cancer, HLA-G can be expressed by the tumor cells and/or by the infiltrating APCs (monocytes/macrophages/DC). Myeloid cells constitutively express both ILT2 and ILT4 HLA-G receptors, and are efficiently inhibited by this molecule (23). Following exposure to HLA-G, APC lose their capability to stimulate T cells (57) and therefore might lose their capability to stimulate iNKT cells as well. Furthermore, HLA-G induces the differentiation of regulatory cells, including regulatory myeloid cells (42, 60). These cells do not support regular T cell activation and might not stimulate iNKT cells either. In order to test this hypothesis, we investigated if HLA-G-expressing tolerogenic DC, called DC-10, were capable of stimulating iNKT cells. DC-10 cells are particularly interesting in our context because they express the same levels of CD1d as mature DC (mDC). Indeed, iNKT cells have been stimulated by artificial APC, expressing costimulatory molecules such as CD80 and CD86. Thus, the lack of stimulation of DC-10 cells compared to mDC could not be explained by lack of costimulation. Generating autologous mDC, DC-10, and ILT2-expressing iNKT to set up and then perform functional studies proved to be a very problematic and uncertain task, which limited the number of experiments we could perform, ultimately preventing our results from reaching statistical significance. However, the tendency was clear and indicates that whereas mDC efficiently activate iNKT cells, HLA-G-expressing tolerogenic DC-10 do not (**Figure 6**). We could not block and prove that HLA-G caused iNKT cell inhibition, for lack of sufficient autologous DC-10 and purified iNKT cells. However, in light of the other results presented here, an HLA-G-mediated inhibition of iNKT following stimulation by αGC-loaded DC-10 is a relevant hypothesis, even though inhibition by IL-10 cannot be excluded, since DC-10 modulatory activity relies not only on the expression of HLA-G but also on the secretion of IL-10 (42). These two mechanisms are actually not mutually exclusive and could act synergically as it has been shown in other contexts (42, 43, 61). It is well known that HLA-G, and especially HLA-G-expressing APCs such as DC-10, not only inhibit the classical function of T cells, but also induce their differentiation into regulatory cells (42). Yamaura et al. demonstrated that IL-10-secreting DCs can induce iNKT cells to produce more IL-10 which will further have an anergic phenotype, and potently inhibit allogeneic CD4^+^ T cell proliferation *in vitro* (62). We do not know the function of DC-10-stimulated iNKT cells, but their differentiation toward a regulatory type is a possibility that should be investigated. Nevertheless, our data show for the first time that iNKT cells are not activated if tolerogenic stimulators are present. This is an important headway in the context of anti-tumor adjuvant therapy using iNKT cells, given that these therapeutic strategies rely on the proper activation of iNKT cells by autologous DC. Thus, because tolerogenic DC or myeloid suppressive cells have been reported in the pathological context of cancer, this might explain the lack of iNKT response in human trials, possibly amplified by iNKT cell differentiation in tolerogenic iNKT cells (62) which would achieve the opposite of the intended goal. Thus, without proper identification, DC-10 cells could very well be present within the autologous myeloid cell population used for iNKT cell stimulation in iNKT-based immunotherapies.

iNKT cells have the ability to rapidly release large amounts of cytokines to link both innate and adaptive immune responses. Hence, iNKT cells possess a potent adjuvant activity: IFN-γ secretion by activated iNKT cells can activate NK cells, mature DC, and prime Ag-specific T cell responses (63) IFN-γ and IL-4 secretions can also contribute to antibody secretion and memory B cell induction (64, 65). Our work demonstrates that HLA-G and myeloid regulatory cells such as HLA-G-expressing DC-10 cells prevent proper activation of iNKT cells by αGC, a mechanism that may very well occur in iNKT cell-based anti-tumor therapy trials and reduce therapy efficiency. More generally, our results emphasize the need for factoring in the functions of ICPs and regulatory cells in iNKT cell-based anti-tumor therapies.

## Data Availability Statement

The raw data supporting the conclusions of this article will be made available by the authors, without undue reservation.

## Author Contributions

C-LW: performed experiments, collected and analyzed data, wrote the manuscript. JC: designed and performed experiments, analyzed data, wrote the manuscript. GA: performed and analyzed experiments. FA: performed experiments. ML: analyzed data, wrote the manuscript. SG: wrote the manuscript. PL-D: manuscript review. JL: designed study, analyzed data, wrote the manuscript. All authors contributed to the article and approved the submitted version.

## Funding

This work was supported by CEA, Invectys and by CIFRE fellowship numbers 2014/0386.

## Conflict of Interest

C-LW,JC, FA, ML and PL-D were employed by Invectys.

The remaining authors declare that the research was conducted in the absence of any commercial or financial relationships that could be construed as a potential conflict of interest.
