# Supplementary material for: Inhibition of iNKT Cells by the HLA-G-ILT2 Checkpoint and Poor Stimulation by HLA-G-Expressing Tolerogenic DC
Source: Front Immunol. 2021 Jan 11;11:608614. doi: 10.3389/fimmu.2020.608614 (PMC7832389; doi:10.3389/fimmu.2020.608614)
Supplement: Supplementary file 1 [file Image_1.pdf]

Supplemental Figure 1

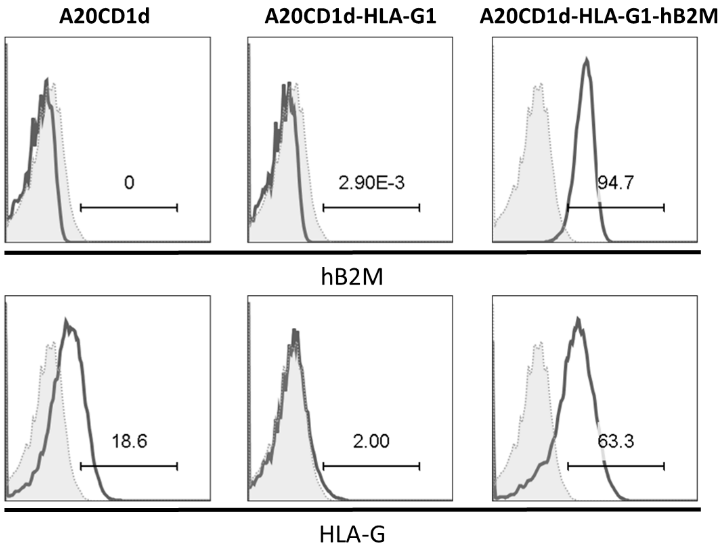

Supplemental Figure 1: HLA-G expression by transduced A20CD1d cells

A20CD1d cells were transduced with HLA-G1 heavy chain onlt (A20CD1d-HLA-G1) or HLA-G1 heavy chain plus human B2M (A20CD1d-HLA-G1-hB2M). hB2M and HLA-G1 expression was verified by flow cytometry. Cell surface HLA-G1 expression was observed only in A20CD1d-HLA-G1-hB2M. Open histograms: staining for the indicated antibodies. Shaded histograms: isotype controls. The percentage of parent population is indicated.
